# Annual public health and economic benefits of seasonal influenza vaccination: a European estimate

**DOI:** 10.1186/1471-2458-14-813

**Published:** 2014-08-07

**Authors:** Emmanuelle Preaud, Laure Durand, Bérengère Macabeo, Norbert Farkas, Brigitte Sloesen, Abraham Palache, Francis Shupo, Sandrine I Samson, on behalf of Vaccines Europe influenza working group

**Affiliations:** Sanofi Pasteur MSD, 162 av Jean Jaures, Lyon, 69367 France; Sanofi Pasteur, 2, Avenue Pont Pasteur, Lyon, 69007 France; Novartis Vaccines & Diagnostics AG, Lichtstrasse 35, 4056 Basel, Switzerland; GlaxoSmithKline, Rue de l’Institut 89, Rixensart, Belgium; Abbott, C.J. van Houtenlaan 36, 1381 CP Weesp, The Netherlands; Creativ-Ceutical Ltd, The Bank Chambers, Borough High Street, London, SE1 9QQ UK

**Keywords:** Influenza, Public health policy, Vaccines and immunization, Modeling, Epidemiology

## Abstract

**Background:**

Vaccination is currently the most effective means of preventing influenza infection. Yet evidence of vaccine performance, and the impact and value of seasonal influenza vaccination across risk groups and between seasons, continue to generate much discussion. Moreover, vaccination coverage is below recommended levels.

**Methods:**

A model was generated to assess the annual public health benefits and economic importance of influenza vaccination in 5 WHO recommended vaccination target groups (children 6 – 23 months of age; persons with underlying chronic health conditions; pregnant women; health care workers; and, the elderly, 65 years of age) in 27 countries of the European Union. Model estimations were based on standard calculation methods, conservative assumptions, age-based and country-specific data.

**Results:**

Out of approximately 180 million Europeans for whom influenza vaccination is recommended, only about 80 million persons are vaccinated. Seasonal influenza vaccination currently prevents an annual average of between 1.6 million and 2.1 million cases of influenza, 45,300 to 65,600 hospitalizations, and 25,200 to 37,200 deaths. To reach the 75% vaccination coverage target set by the EU Council Recommendation in 2009, an additional 57.4 million person would need to be vaccinated in the elderly and other risk groups. By achieving the 75% target rate set in EU-27 countries, average annual influenza- related events averted would increase from current levels to an additional +1.6 to +1.7 million cases, +23,800 to +31,400 hospitalization, +9,800 to +14,300 deaths, +678,500 to +767,800 physician visits, and +883,800 to +1,015,100 lost days of work yearly. Influenza-related costs averted because of vaccination would increase by an additional + €190 to + €226 million yearly, in vaccination target groups.

**Conclusions:**

Full implementation of current influenza vaccination recommendations of 75% vaccination coverage rate (VCR) in Europe by the 2014–2015 influenza season could immediately reduce an important public health and economic burden.

## Background

### The global burden of influenza

Globally, the annual attack rate with influenza viruses ranges between 5 to 10% in adults and 20 to 30% in children [1]. Groups at particular risk of severe influenza include pregnant women, children aged <5 years, the elderly (65 years), and individuals with underlying health conditions. Health care workers are at increased risk of exposure and, if not vaccinated, may be a source of virus transmission, particularly in patient care settings. The WHO estimates that 3–5 million cases of severe influenza illness occur every year resulting in 250,000-500,000 deaths worldwide, with most influenza deaths occurring among adults over 65 years of age [2].

### The burden of influenza in Europe

In Europe, a rough estimate puts the average annual excess deaths from seasonal influenza at 38,500 with considerable seasonal variation [3]. In a sample of European countries influenza mortality rates ranged from around 57.05/100,000 in Spain (1999 and 2005) [4] to 160/100,000 in Slovakia (1982–2000) [5]. The impacts on healthcare costs and productivity are substantial [6]. Ryan et al. estimated that 100% vaccination coverage in risk groups in France, Germany, Italy, Spain, and the UK would result in a savings of €1.59 billion in reduced hospitalizations alone [7]. In 2012–2013, the influenza season in Europe was unusually long, peaking in week 5 of 2013 and ending with a late rise in influenza B cases in many countries [8, 9]. Based on data from 13 European countries or regions, excess mortality for the winter was higher than in the previous 3 influenza seasons, especially in the elderly (65 years) [9]. Influenza-related GP visits in France were approximately twice the number from the previous season (10.2 million vs. 3.5 million) and 1 out of 3 children were affected [10]. The impact on health spending was also significant with one estimate putting the cost of influenza in France at € for doctors’ fees, pharmaceuticals, and compensation for sick-days for the month of January 2013 alone and €220 million for the season by the end of March 2013 [11, 12]. The total impact of an influenza epidemic (including direct and indirect costs) in industrialised countries has been estimated to reach € [13]. Extrapolating from the estimated costs of the 1996–1997 influenza epidemic in Germany (approximately € million), and a French estimate of an annual € million in costs, the costs in the EU would range from €6 billion to €14 billion annually.

### The importance of influenza vaccination

Vaccination is currently the most effective means of preventing influenza infection. Inactivated seasonal influenza vaccines were first licensed in 1945 [14]. Now, several influenza vaccines, including live attenuated or inactivated quadrivalent vaccines in conventional or alternate delivery forms, and adjuvanted vaccines are available. The World Health Organization’s (WHO) position on currently licensed influenza vaccines is that they are safe and efficacious and can prevent significant annual morbidity and mortality [1]. Likewise, the European Centre for Disease Prevention and Control (ECDC) strongly advocates the use of currently licensed influenza vaccines among those for whom they are recommended [15]. Influenza vaccination strategies in Europe typically target groups at greater risk of complications. As such, the EU Council has recommended that member states achieve vaccination coverage rates (VCRs) of 75% in the elderly and, if possible, in other risk groups, and health care workers by the 2014–2015 influenza season [16]. However, vaccination coverage remains suboptimal.

Yet evidence of vaccine performance, and the impact and value of seasonal influenza vaccination across risk groups and between seasons, continue to generate much discussion. Despite the Council recommendation [16], VCRs in Europe have not increased since 2009 and there is evidence of declining trends across all EU member States [17, 18]. This is in part because comparable and consolidated evidence is scarce.

### What our study addresses

We generated a model to assess the public health benefits and economic importance of influenza vaccination in the 5 WHO recommended vaccination target groups (children 6 – 23 months of age; persons with underlying chronic health conditions; pregnant women, health care workers; and, the elderly, 65 years of age) [1] in 27 countries of the European Union (Austria, Belgium, Bulgaria, Cyprus, Czech Republic, Denmark, Estonia, France, Finland, Germany, Greece, Hungary, Ireland, Italy, Latvia, Lithuania. Luxembourg, Malta, Netherlands, Poland, Portugal, Romania, Slovakia, Slovenia, Spain, Sweden, UK)(EU-27), using available values for vaccine efficacy or effectiveness and available vaccine coverage rates. Our assessment included an evaluation of the potential benefits of vaccination at the EU Council recommended 75% vaccination coverage level.

## Methods

Our approach was adapted from the model published by Ryan et al. who estimated the potential benefits from seasonal influenza vaccination in Europe with 100% vaccination coverage [7]. Our model estimations were based on standard calculation methods, conservative assumptions, age-based and country-specific data. We estimated the number of eligible person in different risk groups who would need to be vaccinated to achieve the 75% vaccination coverage target in 27 EU countries. The numbers of avertable influenza-related events and the associated offset costs were estimated in 8 countries (the UK, Germany, Italy, Spain, France, Poland, Slovakia, Sweden) and extrapolated to the EU-27 at current and at 75% VCR. Different values for efficacy and effectiveness of influenza vaccine were used to test the sensitivity of these parameters on the results.

### Estimates

#### Estimating the vaccination gap to achieve 75% vaccination coverage

We estimated the vaccination coverage gap for each of 27 countries, by target group. This was achieved by subtracting the number of people actually vaccinated for influenza in each country from the number of people potentially eligible for influenza vaccination under the 75% target in each target group:


Where *V*_*c,r*_ is the number of people that are vaccinated against influenza virus in priority group *r* living in country *c* (according to the official sources) per year; *V*_*c,r(75%)*_ is the number of people that are expected to be vaccinated against influenza virus, according to 75% target in each country and priority group, per year.

Target groups considered, using the EU Council and WHO 2012 recommended groups definitions, were: children 6 – 23 months of age; persons with underlying chronic health conditions (chronic respiratory conditions, cardiovascular disease, diabetes, renal disease, suppressed immunity, lung cancer, and liver disease); pregnant women; health care workers; and, the elderly (65 years of age for all countries except Germany where 60 years was used).

#### Estimating avertable burden of seasonal influenza

The public health burden of influenza in this study was defined by the following attributes: the numbers of influenza cases, physician visits, hospitalizations, lost days of work, and deaths. Avertable economic burdens were assessed by the costs of general practitioner (GP) visits, hospitalizations, lost days of work, and total costs avoided. We refer to these attributes individually as influenza-related events.

To minimize the impact of methodological bias, distinct estimates were calculated for the numbers of influenza-related events avoided using effectiveness or efficacy values. Effectiveness values reflect actual performance of the vaccine, under programmatic conditions, against laboratory confirmed flu cases.

Efficacy values provide the best quality evidence because they are generated from randomised controlled trials measuring incidence of laboratory-confirmed influenza in vaccination and placebo groups, but the results are scarce for some populations.

Avertable influenza-related events were then estimated by applying the country-specific annual incidence rate of the influenza related event to the unvaccinated eligible population and multiplying the effectiveness value of the vaccine for each target group.


Where *V*_*c,r*_ and *V*_*c,r(75%)*_ have the same meaning as in formula above; *E*_*r*_ is effectiveness of vaccine in preventing influenza cases for priority group *r*; *I*_*cr*_ is the priority group and country-specific incidence of influenza in unvaccinated population.

This method was used to estimate the number of avertable influenza-related events at existing VCRs, and at a 75% coverage rate.

To account for the fact that available epidemiological information was collected in countries where significant proportions of individuals are protected against influenza because of vaccination, the expected rate of influenza-related events in the unvaccinated was calculated as:

The observed rate of influenza-related events in the vaccinated/(1 - Vaccination Coverage × Vaccination Effectiveness).

The cost of avertable influenza-related events was estimated by applying a country-specific unit cost to the total number of influenza related events estimated.

### Data sources

European and especially local data sources are scarce and not available for each country, by season and by specific populations. When needed, extrapolations were done and are described below.

### Vaccination coverage rates (VCR)

VCR data used came from the ECDC (VENICE survey) influenza vaccination surveys, if available, to ensure methodological consistency [19–21]. When information for specific target groups was incomplete, alternate sources were used [22–29]. Most of the data was available for children, the elderly and health care workers. For missing data, extrapolation from other countries was performed by using an average VCR for all countries in a given target group where the VCR information was available. Where data was missing for pregnant women, the general population VCR values – where available - were adopted. This information was available for 12 countries. The values for HCW VCR were adopted for 7 more countries whilst the rest were extrapolated from elsewhere. The VCR for people with chronic diseases group was available for 16 countries .When VCR for this group was missing, the VCR for the elderly from the same country was used (which was always available).

In all cases, only seasonal VCRs for the regular influenza seasons - and never on pandemic vaccine coverage – were collected. Generally, data from the latest year was taken, unless it was from a pandemic year. However, if the only available VCR was from the pandemic year, it was also acceptable and used, as long as it was for seasonal vaccine coverage. The range of years from which data was extracted was for the 2003–2004 to 2009–2010 influenza seasons, the most up-to-date at the time of the analysis

#### Vaccine effectiveness

Effectiveness values were taken from a large case–control study conducted by the US Centers for Disease Control and Prevention (CDC) in the 2010–11 influenza season enrolling 1,048 cases aged 6 months and up, and in 3,768 controls [30] (Table 1). This study was selected as the method used was very strong and it provides recent estimates of vaccine effectiveness in all age groups, in the absence of a single equivalent European source. These values were supplemented with lower and upper values of effectiveness collected from I-MOVE, a European network supported by ECDC that measures the effectiveness of influenza vaccines against laboratory confirmed influenza. The data came from the 2010–2011 [31] and 2011–2012 seasons [32]. These values were selected to reflect the differences that can be observed across flu seasons. It was assumed that the incidence of all influenza- related events would be reduced in the same proportion as the probability of laboratory-confirmed influenza (LCI).

**Table 1:** **Baseline vaccine effectiveness and efficacy of TIV with upper and lower limits, by target group**

Vaccination target group	Baseline vaccine effectiveness (%)	Lower limit of vaccine effectiveness (%)	Upper limit of vaccine effectiveness (%)	Baseline vaccine efficacy (%)	Lower limit of vaccine efficacy (%)	Upper limit of vaccine efficacy (%)	RCTs used for efficacy
**6 – 23 months**	59 [30]	19.4 [32]	65.7 [31]	45 [33]	25	65	2 studies, 786 recruits (525 vaccinated 261 placebo)
**Healthy, 2–64 years (health care workers and pregnant women)**	51 [30]	41.3 [31]	63.3 [32]	61 [34]	48	70	17 studies 31,325 recruits (16,213 vaccinated 15,112 placebo)
**Underlying chronic conditions, 2–64 yrs**	51 [30]	41.3 [31]	63.3 [32]	61 [34]	48	70	17 studies 31,325 recruits (16,213 vaccinated 15,112 placebo)
**Elderly**	39 [30]	15.1 [32]	59.9 [31]	58 [35]	34	73	3 studies, 2217 recruits (1,110 vaccinated 1,107 placebo)

#### Vaccine efficacy

Efficacy values were taken from Cochrane systematic reviews comparing inactivated trivalent influenza vaccines with placebo [33–35] (Table 1). Only values of efficacy for LCI, from randomised controlled trials (RCTs), were used. It was assumed that the incidence of all influenza-related events would be reduced in the same proportion as the probability of LCI. For the upper and lower limits used, we chose the confidence intervals on the meta-analysis. For the group of children 6 – 23 months, the upper and lower confidence limits were very wide (-69 – 82) [33] so these were substituted with 25 and 65 respectively: the lower limit was chosen to be consistent with the difference between the mean and lower limit in the elderly group, while the upper limit was chosen to be comparable with the efficacy values observed in healthy adults.

#### Influenza attack rates

The influenza target-group-specific attack rates were derived from the weighted average of influenza incidence in the placebo arms of 29 Cochrane reviewed RCTs worldwide: 9 articles for children; 2 for the elderly; and, 18 for healthy adults. It was assumed that the age-specific average annual rate of true influenza was equal to the weighted average of influenza incidence in the placebo arm. The same age-specific attack rates were applied across all countries (Table 2).

**Table 2:** **Age specific data used in the model for each country or for all EU-27**

Data used for modeling	Vaccination target group	Germany	France	Italy	Spain	UK	Poland	Slovakia	Sweden	EU-27
**Influenza attack rate**	6-23 months	19.10%	19.10%	19.10%	19.10%	19.10%	19.10%	19.10%	19.10%	19.10%
	Healthy, 2–64 years (health care workers and pregnant women)	3.64%	3.64%	3.64%	3.64%	3.64%	3.64%	3.64%	3.64%	3.64%
	Underlying chronic conditions, 2–64 yrs	3.64%	3.64%	3.64%	3.64%	3.64%	3.64%	3.64%	3.64%	3.64%
	Elderly	4.91%	4.91%	4.91%	4.91%	4.91%	4.91%	4.91%	4.91%	4.91%
**Influenza-related GP visits rate**	6-23 months	5.99%	5.20%	5.97%	3.45%	7.21%	2.97%	5.40%	2.05%	
	Healthy, 2–64 years (health care workers and pregnant women)	2.35%	2.04%	2.25%	1.35%	1.18%	1.17%	2.34%	2.55%	
	Underlying chronic conditions, 2–64 yrs	2.35%	2.04%	2.25%	1.35%	1.18%	1.17%	2.34%	2.55%	
	Elderly	1.58%	1.37%	0.89%	0.94%	0.60%	0.78%	0.58%	1.01%	
**Influenza-related hospitalizations/100,000**	6-23 months	127.90	73.70	76.90	67.60	131.10	134.70	205.50	106.10	
	Healthy, 2–64 years (health care workers and pregnant women)	12.70	7.30	7.60	6.70	4.90	13.30	20.30	10.50	
	Underlying chronic conditions, 2–64 yrs	34.30	19.80	20.60	18.10	17.90	36.10	55.10	28.50	
	Elderly	179.50	103.40	107.90	94.90	130.00	189.00	288.40	148.90	
**Influenza-related mortality/100,000**	6-23 months	1.62	0.80	0.83	1.09	1.28	2.02	3.07	1.93	
	Healthy, 2–64 years (health care workers and pregnant women	0.00	0.00	0.00	0.00	0.00	0.00	0.00	0.00	
	Underlying chronic conditions, 2–64 yrs	4.90	4.86	5.06	2.31	3.87	6.10	9.28	5.84	
	Elderly	84.70	84.00	87.50	57.05	66.90	105.00	160.55	101.00	

#### Epidemiological data

Since methodological differences exist in EU-wide national influenza surveillance systems, a sample of 8 countries (France, Germany, the UK, Spain, Italy, Sweden, Poland and Slovakia) (EU-8) was selected and values were extrapolated to the remaining 19 countries (Table 2) based on 4 geographical regions. The average values for epidemiological burden data from France, Germany and the UK were applied to Austria, Belgium, Ireland, Luxembourg, the Netherlands; values for Sweden were applied to Denmark and Finland; average values for Italy and Spain were applied to Cyprus, Greece, Malta, Portugal, Slovenia; and, average values for Poland and Slovakia were applied to Bulgaria, Czech Republic, Estonia, Hungary, Latvia, Lithuania, Romania. The EU-8 countries represent more than 70% of the total population of the EU-27, and 7 of these account for 72% of the vaccination gap. Country-specific missing data were usually replaced by similar data from neighboring countries or from another risk group in the same country. For instance, when country specific information on influenza-related mortality rates were not available, rates were extrapolated from Czech Republic for Poland and Slovakia, and from Norway for Sweden. For missing data in the underlying chronic health conditions group, the coverage rate for the elderly from the same country was applied. For missing data in other target groups, the average coverage rates from all other countries were applied.

Epidemiological data varied by country but was usually available for years ranging from 2000 – 2011, except for mortality rates which were available for years 1969–2009, and GP visits for which the range was 1986 – 2011.

#### General Practitioner (GP) visits

Data on influenza-related GP visits was captured from country-specific surveillance. Only the UK had age-specific data on influenza related GP visits. Age-specific estimates in other countries were based on the proportion of age-specific influenza-like-illnesses (ILI) rates. In countries where information was available on ILI-related GP visits (rather than on influenza), it was assumed that ILI incidence was 3.65 times higher than influenza incidence. The constant is the ratio of ILI to influenza rates from a Cochrane Review that pooled data from 12 RCT studies (weighting for sample size) and included a total of 13,242 healthy adults reporting both incidence of ILI and influenza in their placebo arms [34].

#### Hospitalizations

Hospitalizations avoided were estimated using excess all-cause influenza-related hospitalization rates. For the UK, data from Pitman et al. [36] were used whereas in the other countries rates were estimated based on age-specific rates of all-cause influenza-related hospitalizations from the Netherlands [37]. Age-specific estimates from the Netherlands were extrapolated to other European countries with adjustment according to the ratio of the annual incidence of hospitalisations with a primary diagnosis of acute upper respiratory infection and influenza in each country over the incidence in the Netherlands. Estimates of annual incidence of hospitalization with a primary diagnosis of upper respiratory infections and influenza were sourced from Eurostat [22].

#### Mortality

Data on excess all-cause influenza-related mortality, for all age groups combined, was available for Germany [38], Italy [39] and the UK [40]. Influenza-related mortality for all age groups combined using country-specific methods by authors in the literature were then derived from these. In the absence of age-specific data, age-specific mortality rates were calculated assuming the distribution of influenza-attributable deaths between age groups was similar to the distribution reported in Norway [41], the only European source of age-specific data identified. The influenza-related mortality rate among people with underlying chronic health conditions was assumed to be the same as for people aged 45–65 in the general population, and mortality was assumed to be 0 amongst pregnant women (out of pandemic period) and healthcare workers. For Poland, Slovakia and Sweden, where data on influenza-related mortality rates were not available, rates were extrapolated from the Czech Republic, Poland and Norway respectively, with adjustment according to the age-specific relative rates of hospitalization between countries. This was done to moderate any difference in magnitude between the rates of influenza-related events between two countries. Similarly for France and Spain, influenza-attributable mortality rates were extrapolated from Italy, with adjustment according to age-specific ratios of annual incidences of hospitalizations with primary diagnoses of acute respiratory infection and influenza between those countries and Italy. The data for Italy were from Rizzo et al. [39].

#### Lost work days

These were estimated to occur both for parents attending to sick children and for illness amongst working adults. The number of potentially preventable missed working days for parents who take care of sick children was estimated as the number of potentially preventable GP visits associated with influenza for children under 2, multiplied by the employment adjustment (since in some cases parents will not be working, and therefore no work days will be missed), the number of days missed in case of absence, and the probability of absence among cases with GP consultation. OECD statistics on employment rates were used, representing the population of working age (15–64 years). The expected number of days taken off work for adults in this study was estimated as the product of the probability of taking time off (91% for sickness from influenza), [42] and the average duration of time off for influenza sickness, 4 days [42, 43]. This was applied to all countries and for all adult target groups.

#### Economic data

Country-specific unit costs were available for 8 countries (Germany, France, Italy, Spain, the UK, Poland, Slovakia, Sweden) and extrapolated to the remaining 19 by matching each against one of the EU-8 reference countries judged to be most similar in terms of health system: Germany served as a reference for Austria; France served as a reference for Belgium, Luxembourg, and the Netherlands; Slovakia served as a reference for Bulgaria, Czech Republic, Estonia, Hungary, Latvia, Lithuania, Romania, and Slovenia; Spain served as a reference for Cyprus, Greece, Malta, and Portugal; Sweden served as a reference for Denmark and Finland; and the UK served as a reference for Ireland. Italy and Poland did not serve as references.

Estimates were made for the costs of GP visits, hospitalizations and lost wages.

#### GP costs for influenza-related visits

These were country-specific and estimated to be the same for all age and risk groups except in France where the cost of physician visits is higher for children than for adults [44–51].

#### Hospitalization costs for influenza related illness

Hospitalization costs were obtained from diagnostic related group (DRG) tariffs or hospitalization statistics in different countries, except for Poland, where a cost from the literature was used [50]. For each age group, a weighted average cost was calculated, with weights equal to the proportions of different diagnoses (upper respiratory tract infections (URTI); lower respiratory tract infections and pulmonary disease (LRTI); cardiovascular diseases (CVD) and other complications) as reported by Jansen et al. [37].

#### Lost wages

To estimate the total value of work-related monetary losses, the number of potentially preventable days missed from work per country was multiplied by the monetary value of each missed working day using OECD statistics [47].

#### Total offset costs

The total cost offsets for EU-27 were calculated by combining the costs avoided per country, by risk group, for GP visits, hospitalization and for lost wages due to absenteeism from work. Total costs were estimated for current VCR and 75% VCR for all risk groups. Estimates were made using values for both vaccine effectiveness and vaccine efficacy.

## Results

### Vaccination coverage gaps

**Figure 1:**
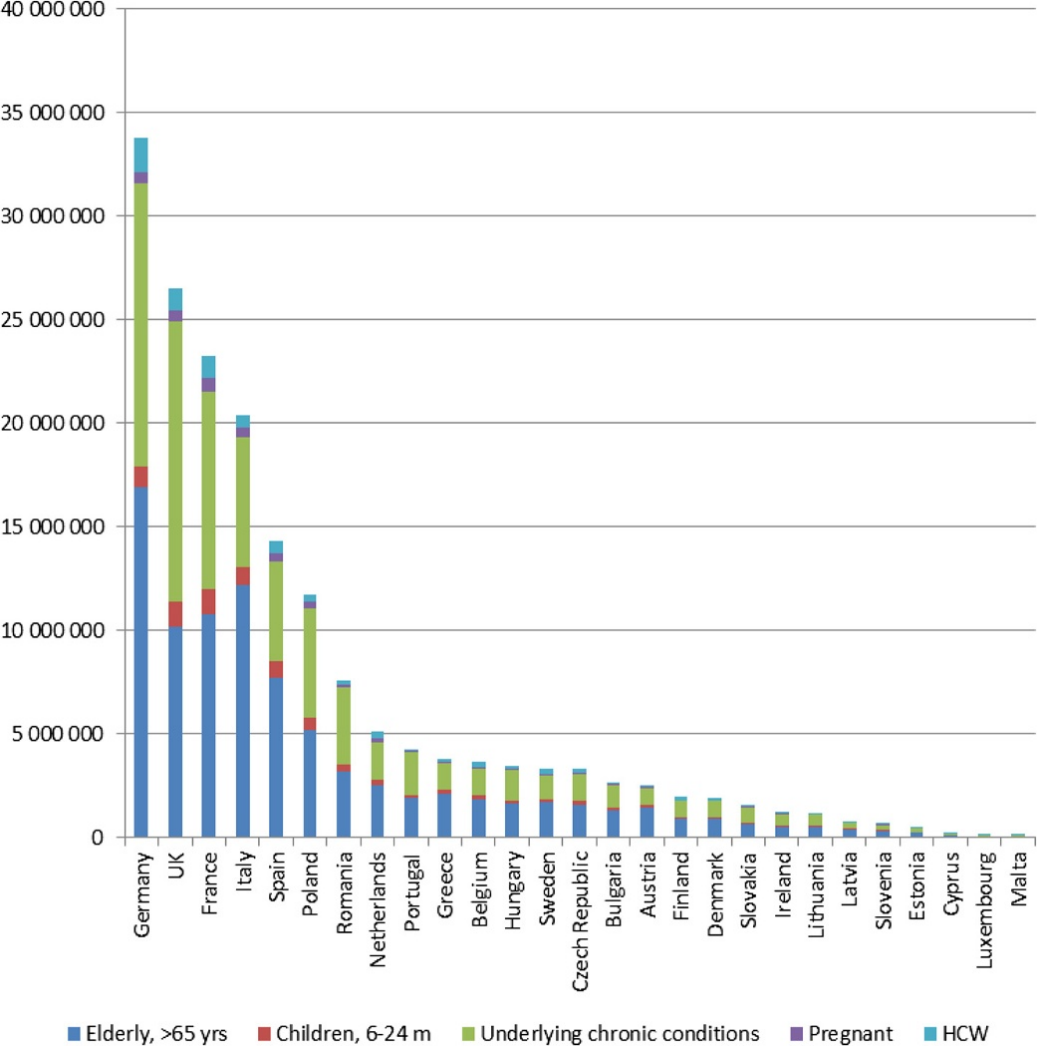
**Population for whom influenza vaccination is recommended in 27 EU countries.**

Target groups for influenza vaccination represent 36% of the EU-27 population, amounting to approximately 180 million persons eligible for vaccination. The break down by age-specific groups and by country is shown in Figure 1. Among recommended target groups for influenza vaccination, the elderly (persons over 65) account for 48% of all targeted groups, whereas 41% are persons with underlying chronic health conditions (2 to 64 years). Children, healthcare workers, and pregnant women represent the remaining 5%, 4% and 2% respectively.

**Figure 2:**
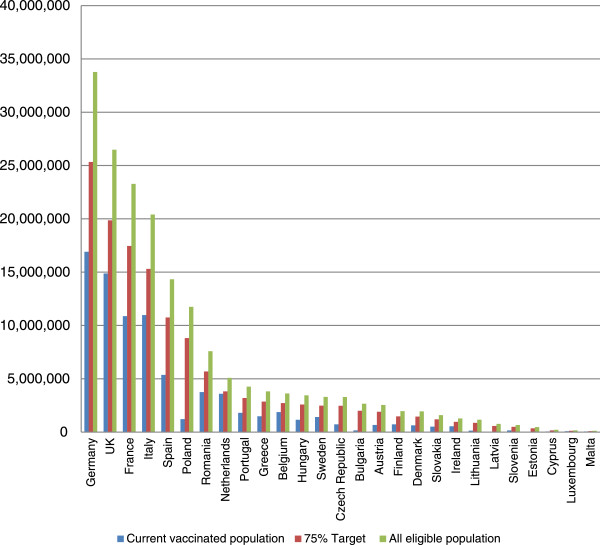
**Actual number versus 75% VCR versus 100% VCR vaccinated in 27 EU countries.**

Only about 80 million persons (44% of the eligible) in EU-27 are vaccinated annually. None of the EU-27 countries has achieved the EU Council’s target of 75% coverage. Most countries are considerably below targets, with some countries, such as Estonia, achieving as little as 1% VCR in the elderly target group.To reach a 75% vaccination coverage target, an additional 57.4 million people would need to be vaccinated (Figure 2). Fifty percent of the vaccination gap is constituted by persons with underlying chronic health conditions (2 to 64 years), 29% by the elderly, 9% by children 6–23 months, 7% by healthcare workers, and 5% by pregnant women. However, the elderly represent the largest target group for seasonal influenza vaccination (47% of the eligible population) and have the highest rates of influenza- related complications including death. Therefore, the elderly constitute a particularly important target group for improved vaccination coverage, especially in the countries where coverage in this target group is very low, such as in Eastern Europe.

### Estimated averted burden with current vaccination coverage rates

The vaccination target-specific estimates for averted influenza-related events at current VCR in EU-8, representing more than 70% of the population of the EU-27, are shown in Table 3. Using efficacy values, 70% of cases averted were in the elderly, 25% in persons with underlying chronic health conditions, and about 3% in children, 1% in healthcare workers, and <1% in pregnant women. For GP visits averted, the elderly accounted for 57% and persons with underlying chronic health conditions for 38%. Ninety-two percent of averted hospitalizations and 98% of averted deaths were in the elderly, whereas and 7% and 2% respectively were in persons with underlying chronic health conditions. But for lost work days averted, the chronically ill group accounted for 92%.

**Table 3:** **Vaccination target-specific averted influenza-related events, in EU-8 countries (France, Italy, Spain, UK, Poland, Slovakia, Sweden and Germany) using efficacy values at current VCR**

Influenza-related events averted	6 – 23 months	Elderly	Chronically ill	Healthcare workers	Pregnant women	Totals
cases (%)	55,626 (3.3)	1,178,452 (70.0)	418,050 (24.8)	21,046 (1.2)	11,026 (0.7)	1,684,200
GP visits (%)	17,585 (2.4)	423,686 (57.1)	280,906 (37.9)	13,342 (1.8)	6,604 (0.9)	742,123
Hospitalizations (%)	333 (0.6)	50,706 (92.5)	3,704 (6.8)	60 (0.1)	29 (0.1)	54,832
Deaths (%)	3 (0.0)	30,238 (97.7)	705 (2.3)	0	0	30,946
Days of work lost (%)	9,492 (1.6)	0	558,516 (92.0)	26,713 (4.4)	12,650 (2.1)	607,371

Extrapolating to EU-27 at current VCR (Table 4), using vaccine effectiveness and vaccine efficacy values respectively, we estimated that seasonal influenza vaccination currently prevents an average of between 1.6 million and 2.1 million cases of influenza each year. Average annual GP visits, hospitalizations, deaths, and lost days of work averted were estimated at: 701,200 to 916,000; 45,300 to 65,600; 25,200 to 37,200; and, 715,400 to 844,700, respectively. The corresponding costs avoided for GP visits, hospitalizations and lost days of work were estimated at about: €22 million to €29 million; €131 million to €190 million; and, €96 million to €113 million respectively.

**Table 4:** **Estimated public health and economic impacts of seasonal trivalent influenza vaccination extrapolated to EU-27 countries**

Influenza-related event	Numbers and costs currently averted at existing VCR (lower, upper limit)			
	Estimates using effectiveness values		Estimates using efficacy values	
	Numbers of events averted	Costs averted	Number of events averted	Costs averted
**Cases of influenza**	1.6 million (0.82; 2.2)	-	2.1 million (1.3; 2.6)	-
**Influenza-related mortality**	25,161 (10,092; 38,390)	-	37,165 (21,986; 44,667)	-
**Influenza- related GP visits**	701,234 (417,680; 958,010)	€22 million (13; 30)	915,997 (620,643; 1,110,498)	€29 million (20; 35)
**Influenza-related hospitalization**	45,325 (19,440; 67,970)	€131 million (56;197)	65,593 (39,536; 82,036)	€190 million (141; 238)
**Lost days of work**	715,428 (568,878; 881,851)	€96 million (76; 118)	844,748 (661,843; 973,075)	€113 million (88; 130)
**All Influenza-related costs**	-	€248 million (145; 345)	-	€332 million (222; 403)

### Estimated benefits of reaching 75% vaccination coverage rate

Using vaccine effectiveness and vaccine efficacy values respectively, we estimated that increasing the VCR to 75% in EU-27 countries would avert an average additional +1.6 million to +1.7 million cases yearly over currently averted cases.

Additional avertable average annual GP visits, hospitalizations, death and lost days of work were estimated at: +678,500 to +767,800, +23,800 to +31,400, +9,800 to +14,300, and +883,800 to +1,015,100 respectively. The corresponding additional costs averted were estimated at: + €20 million to + €23 million for GP visits, + €57 million to + €75 million for hospitalizations, and + €112 million to + €128 million for lost days of work. Total influenza-related costs would be offset by + €190 million to + €226 million yearly, in recommended vaccination target groups (Table 5).

**Table 5:** **Estimated additional (+) impacts of seasonal trivalent influenza vaccination extrapolated to EU-27**

Influenza-related event	Numbers and costs averted additional (+) to currently averted events if VCR is increased to 75% (lower, upper limit)			
	Estimates using effectiveness values		Estimates using efficacy values	
	Number of events averted	Costs averted	Number of events averted	Costs averted
**Cases of influenza**	+1.6 million (0.85; 2.0)	-	+1.7 million (1.1; 2.1)	-
**Influenza-related mortality**	+9,843 (4,185; 14,831)	-	+14,342 (8,620; 17,939)	-
**Influenza- related GP visits**	+678,482 (424,321; 849,284)	+€20 million (13; 25)	+767,787 (543,913; 935,732)	+€23 million (16; 28)
**Influenza-related hospitalization**	+23,792 (11,199; 33,525)	+€ 57 million (28; 81)	+31,350 (19,545; 39,254)	+€75 million (47; 94)
**Lost days of work**	+883,750 (671,094; 1,081,253)	+€112 million (85; 137)	+1,015,145 (783,557; 1,184,489)	+€128 million (99;150)
**All Influenza-related costs**	-	+€190 million (125; 243)	-	+€226 million (162;272)

The analysis from the EU-8 countries shows that raising VCR would increase the number of cases averted most in the group of persons with underlying chronic conditions (+38%) and in the 6–24 month group (+27%). For GP visits and lost days of work, 52% of visits and 76% of lost days of work are avoided in persons with underlying chronic conditions. However, the majority of avertable hospitalizations and deaths occur for the elderly (70% and 91% respectively).

## Discussion

Our model estimated that seasonal influenza vaccination in Europe currently averts each year between 1.6 to 2.1 million cases, and prevent between 25,200 and 37,200 deaths, with corresponding significant impact on hospitalisations, GP visits and costs avoided (between €248 and €332 million saved annually). The number of avertable events was within a same range regardless if effectiveness or efficacy values were used and the benefits were substantive even at the lowest estimated values, supporting the strong public health interest of influenza prevention.

Raising the VCR from current levels in Europe to the recommended 75% target, in all WHO- recommended vaccination target groups, would avert an additional average annual +1.6 to +1.7 million cases, +678,482 to +767,787 physician visits, +23,793 to +31,350 hospitalization, +9,843 to +14,342 deaths, and +883,750 to 1,015,145 lost days of work. Influenza-related costs would be offset by an additional + €190 to + €226 million over currently averted costs.

While the purpose of our study was not to estimate the net budget impact of influenza vaccination, our results are complementary to the WHO position [1] and to recent papers demonstrating that influenza vaccination is cost effective or even cost-savings in EU countries [52, 53]. Estimating the cost of increasing vaccination coverage will be very specific to the healthcare system. In some countries, increasing coverage using existing infrastructure will require minimum investments compared to the net public health and economic savings generated. In other countries, particularly where vaccination coverage is very low, a greater initial investment will be necessarily but it will also improve access to preventive services for the most vulnerable. Where additional resources are required to achieve the recommended 75% VCR, countries should consider who is currently covering the curative costs of influenza because those payors will have the greatest incentive to invest in prevention.

It has been argued that increased effectiveness/efficacy of seasonal influenza vaccines is needed [15]. We estimated the impact of a hypothetical vaccine with 80% effectiveness/efficacy compared to the impact of increasing vaccination coverage with current trivalent influenza vaccines to 75%. Overall, the impacts were similar but this does not consider the possible impact that increasing vaccine effectiveness might have on vaccination acceptance. Furthermore, there are challenges associated with achieving 80% vaccine effectiveness, particularly in the elderly with immuno-senescence, and with seasonal strain drift, and with vaccine strain mismatches. Further vaccine research and development is ongoing to overcome the associated challenges, but in the meantime increasing coverage with existing vaccines remain the best present solution to fight against a preventable disease.

In the current analysis the following limitations apply: a simplified approach was used which did not account for the indirect benefits of influenza vaccination linked to herd immunity. This underestimates the potential benefits of influenza vaccination. The model also only estimated some of the indirect costs (from work days lost) and some of the direct costs (GP visits and hospitalisations), providing underestimated figure of the possible economic benefits.

Additional limits relate to lack of data in EU and country-specific data and the methods used to account for missing data. The choice of estimating average values across seasons was clearly driven by the absence of season specific data and aimed at reflecting an estimate of influenza vaccination benefits across years. Different sources were taken for effectiveness and efficacy to account for variability between seasons and sources, and results were described accordingly however they might not be relevant for seasons with extreme viral circulation or vaccine mismatch. The model also did not account for the impact of increasing vaccination coverage which can result in a decreasing risk in the unvaccinated and decrease vaccine effectiveness, but this impact is expected to be small.

To deal with local data scarcity, country-specific missing data were usually replaced by similar data from neighboring countries or from another risk group in the same country, and for missing data in other target groups, the average coverage rates from all other countries were applied. This may lead to an overestimate of current VCR in the chronically ill group, for these countries, meaning that annual benefits from seasonal vaccination might be overestimated but meaning also that the benefits from increasing VCR and raising EU Council recommendation are underestimated. On the other hand, the use of incidence data on influenza-related events from surveillance networks in countries may underestimate the actual number of GP visits, since not all events will have been detected.

Kastova et al. [54] conducted a similar study to ours in the US. They collected surveillance data from six influenza seasons in the US. They defined impact as both the number of averted outcomes and the prevented disease fraction. Presenting impact as the prevented disease fraction allows to control for the relative severity of different seasons. They showed that a greater fraction of disease was prevented as greater fractions of the population were vaccinated.

As in our model, Kastova et al. used an annual vaccine effectiveness estimate based on the range of available vaccine effectiveness estimates in the literature for each season. They also performed sensitivity analyses around their assumptions for missing data to account for uncertainty. In their study, influenza illnesses averted by vaccination ranged from approximately 1.1 million to 5 million (or 357 – 1,641 per 100,000 population) during a season while the number of averted hospitalizations ranged from a 7,700 to 40,400 (2 – 13 per 100,000). This compares with an estimate from our study of approximately 311 – 409 averted cases and 9 – 13 averted hospitalizations per 100,000 population in Europe. Our findings are corroborated by those of Kastova et al. who found that influenza vaccination programs produce a substantial health benefit in terms of averted cases, clinic visits, hospitalizations, and deaths. Both Kastova et al. and our model support the need for improvements in vaccination coverage among non- elderly persons and improvements in vaccine effectiveness among the elderly to improve vaccination program effectiveness.

## Conclusions

Our model reveals that, in spite of currently low vaccine coverage and less than expected vaccine effectiveness, the public health impact and offset costs are important. The results complete evidence of influenza vaccination cost-effectiveness and provide significant figures of annual burden prevented. Both public health and economic benefits from seasonal influenza vaccination can be significantly increased if the 75% vaccination coverage rate is reached: twice as many cases could be prevented and hundreds of thousands of hospitalizations and physicians visits could be avoided, reducing the burden on healthcare systems. In a context of ageing population, crowed healthcare systems, and budget constraints, the economic savings from a reduction in the costs of influenza disease and deaths are important to consider.

To achieve the 2014–2015 EU Council recommendation for seasonal influenza vaccine coverage in Europe, public health officials and the healthcare community should remove the barriers that underpin suboptimal implementation of national/EU recommendations on seasonal influenza vaccination based on the findings from previous studies [55], and find innovative and effective ways to improve access of European citizens to influenza vaccination delivery and health care professionals (such as vaccination at pharmacies or in shopping centers). Our model highlights the need for program options to be designed to better cover specific target groups and for countries to implement evidence-based vaccination policies.

In parallel, the medical community and the vaccine industry should continue to invest in research and development (R&D) to develop novel influenza vaccines. There are over 200 clinical trials ongoing in influenza of which about 25% are in phase I or II, and over 100 biotechnology companies working on influenza [56]. Adjuvanted, intradermal, and nasal spray vaccines are available for the upcoming influenza season and new live attenuated and inactivated quadrivalent influenza vaccines are licensed in the US [57] and EU.

Both increased vaccination coverage and continued development of influenza vaccines are complementary approaches and will better protect European population from seasonal influenza epidemics. Full implementation of current influenza vaccination recommendations could immediately reduce the burden of seasonal influenza infections and increase efficiencies in allocation of health resources and boost economic growth by preventing loss of productivity and preserving health.

Expanding coverage to additional vaccination target groups, as has been done in the US, and more recently the UK, would also increase the benefits. With the successful development of new and improved vaccines, still more benefits can be achieved in the future.
